# An Insight into the Mechanical Properties of Unidirectional C/C Composites Considering the Effect of Pore Microstructures via Numerical Calculation

**DOI:** 10.3390/polym16182577

**Published:** 2024-09-12

**Authors:** Jian Ge, Xujiang Chao, Wenlong Tian, Weiqi Li, Lehua Qi

**Affiliations:** School of Mechanical Engineering, Northwestern Polytechnical University, Xi’an 710072, China

**Keywords:** unidirectional composites, transverse strength, RVE model, pores, failure mechanics

## Abstract

Pores are common defects generated during fabrication, which restrict the application of carbon/carbon (C/C) composites. To quantitatively understand the effects of pores on mechanical strength, this paper proposes a representative volume element model of unidirectional (UD) C/C composites based on the finite element method. The Hashin criterion and exponential degraded rule are used as the failure initiation and evolution of pyrolytic carbon matrices, respectively. Interfacial zones are characterized using the cohesive constitutive. At the same time, periodic boundary conditions are employed to study transverse tensile, compressive, and shear deformations of UD C/C composites. Predicted results are compared with the experimental results, which shows that the proposed model can effectively simulate the transverse mechanical behaviors of UD C/C composites. Based on this model, the effects of microstructural parameters including porosity, pore locations, the distance between two pores, pore clustering, and pore shapes on the mechanical strength are investigated. The results show that porosity markedly reduces the strength as porosity increases. When the porosity increases from 4.59% to 12.5%, the transverse tensile, compressive, and shear strengths decrease by 35.91%, 37.52%, and 30.76%, respectively. Pore locations, the distance between two pores, and pore clustering have little effect on the shear strength of UD C/C composites. For pore shapes, irregular pores more easily lead to stress concentration and matrix failure, which greatly depresses the bearing capacity of UD C/C composites.

## 1. Introduction

Carbon/carbon (C/C) composites are widely applied in the fields of aeronautics and astronautics due to their excellent thermophysical properties, high specific strength, and moduli [1,2,3]. The preparation methods of C/C composites are mainly liquid-phase impregnation/carbonization [4] and chemical vapor infiltration (CVI) [5] techniques, where the latter is the most commonly used method. This method utilizes chemical reactions between gasses to fabricate pyrolytic carbon on the surface of carbon fibers for obtaining densified C/C composites [6,7]. Because of the limitations of the CVI method, some defects, containing vacancy, carbon adatoms, and foreign adatoms, in the pyrolytic carbon will be generated [8], and some pores, i.e., regions uncovered by the pyrolytic carbon, will also be obtained [9]. Generally, the size of the defects in the carbon material is in the range of nanometers, while the size of the pores lies in the range of micrometers. The defects in the carbon material directly affect the properties of the pyrolytic carbon. In this paper, C/C composites consist of carbon fibers, pyrolytic carbon matrices, and pores at the microscale. Experimentally, it has been confirmed that the pore, as an individual component, has a detrimental effect on the mechanical strength of C/C composites [10,11]. Thus, a systematic, quantitative understanding of the effect of pores is beneficial to design C/C composites that meet actual service requirements.

Currently, the investigation methods for composites are divided into three categories: experimental, analytical, and numerical. Owing to being experimentally time-consuming and high-cost, the analytical and numerical predictions have become fashionable. For the analytical method, some semi-empirical equations have been developed, such as the rule of mixture [12], the bridging model [13], and the Rosen and Lo–Chim models [14]. Although these models have high computational efficiency, it is difficult to determine the local stress and strain fluctuations for composites with complex microstructures. Compared to the analytical method, the numerical method is a better choice for dealing with complex microstructures and the local stress and strain field. 

In numerical methods, researchers have performed much work on predicting the mechanical behaviors of composites. Meanwhile, some failure criteria have also been developed based on the observed phenomena, including the Hashin, Tsai–Wu, and Puck rules. The combination of these failure criteria and numerical methods, such as the finite element method, is generally used to evaluate the damage behaviors of composites. For example, Ye [15] predicted the uniaxial stress–strain response and off-axis failure strength of fiber-reinforced epoxy composites based on the Hashin failure criterion. The calculation results agreed with the experimental results. Zhao [16] used the Tsai–Wu equation as the failure initiation criterion for predicting the tensile behavior of epoxy-based laminates. Song [17] adopted the puck criterion to simulate the fiber and matrix failure of composite pressure vessels. Although they have made some advances in the field of the damage of composites, some defects, such as pores, were not further considered.

To understand the effects of pores on the load-bearing capacity of composites, some factors characterizing pores, such as the pore orientation, pore shape, porosity, pore locations, distances between pores, and pore clustering, need to be studied in detail. According to Hyde’s investigation [18], the effect of pore orientations was limited while, the pore shape effects would be amplified as the porosity increased for evaluating the mechanical strength of composites. Vajari [19] found that porosity reduced the transverse tensile and compressive strengths, and pores affected the localization path in unidirectional (UD) composites. In He’s work [20], it was concluded that the stress concentration near pores easily led to matrix damage, and an increase in porosity made the strength of composites decrease significantly. However, these studies pay much attention to the damage behaviors of carbon fiber-reinforced epoxy composites. Unlike epoxy-based composites, C/C composites have very complex microstructures and plenty of irregular pores with a random distribution. Presently, the developed models generally take one or some pore characterization factors to predict the mechanical properties of C/C composites. Chao [21] effectively predicted the elastic properties of C/C composites by approximating pores as polygons using the finite element model. Ali [22] created an image-based finite element model to simulate the stress–strain behavior of 2D woven C/C composites using the X-ray computed microtomography method. Ai [23] and Xu [24] established the mesoscale representative volume element (RVE) model to simulate the mechanical behaviors of 3D orthogonal C/C composites and braided C/C composites by choosing some matrix elements as pores. Despite making some progress in these efforts, systematically analyzing the effects of pores on the mechanical strength of C/C composites is lacking and needs to further be performed quantitatively. 

To quantitatively investigate the effects of pores, the RVE model of the UD C/C composite is proposed to compute the transverse tensile, compressive, and shear strengths in this paper. Based on the Hashin failure criterion and exponential damage evolution law, combining the finite element calculation and subroutine UMAT of the 2020a Abaqus software is programmed to determine the mechanical response of pyrolytic carbon matrices. At the same time, the cohesive constitutive is used to simulate the interfacial deformation. Compared to the experimental results, the proposed RVE model is validated. Finally, the effects of porosity, pore locations, the distance between two pores, pore clustering, and pore shapes are studied based on this model. 

## 2. Experimental Section

The UD C/C composites are prepared using the CVI method [7,25]. Methane serves as the precursor gas, and nitrogen is the diluted gas in the fabrication process. The carbon fiber preform is densified under a temperature of 900~1100 °C and a pressure of 8~12 kPa, and the infiltration time is 80~120 h. It is worth noting that the fiber preform needs to be fixed by the porous graphite plate. For UD C/C composites, the fiber volume fraction is about 40%, and the porosity is determined by the Archimedes method [26]. Equation (1) gives the calculation for porosity.

(1) $$V_{p}=\frac{m_{1}-m_{0}}{m_{1}-m_{2}}$$

where *m*_0_ and *m*_1_ denote the mass of the dried and water-infiltrated sample, respectively; and *m*_2_ is the mass of the sample in the water.

To test the transverse mechanical strength of UD C/C composites, an electronic universal testing machine (CMT5304 made by MTS in America) is employed with a crosshead speed of 0.5 mm/min for tension, compression, and shearing at a room temperature of 25 °C. For this device, the peak load is 30 kN. Before testing, the UD C/C composites are machined into 3~5 rectangular samples for the tension and compression, where the dimensions of samples are 75 × 8 × 4 mm^3^ for the tension and 4 × 4 × 5 mm^3^ for the compression according to ASTM C1275-95 [27] and DIN EN 658-2 [28], respectively. For shearing, the double-notch shear testing standard (ASTM D 3846) [29] is used, where the dimensions are 30 × 4 × 15 mm^3^ with a two-notch distance of 6 mm. 

**Figure 1:**
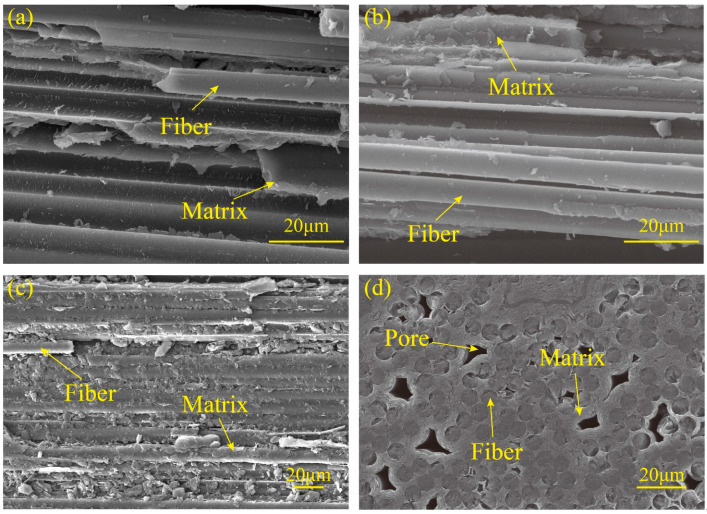
The fracture morphology of UD C/C composites under (**a**) the tensile, (**b**) the compressive, and (**c**) the shear loadings; (**d**) the cross-section of UD C/C composites.

Figure 1a–c presents the fracture morphologies of UD C/C composites after completing the tensile, compressive, and shear tests, respectively, where the scanning electron microscope (SEM) called Tescan Vega3 from Brno in The Czech Republic is used. It is observed that fibers remain intact, the matrix breaks off, and interfacial debonding partially occurs. Therefore, the transverse strength of UD C/C composites is dependent on the matrix and interfacial strength. Figure 1d provides the cross-section morphology of UD C/C composites, where pores exist in the pyrolytic carbon matrices.

## 3. Simulation Models and Methods

This section illustrates details of creating RVE models and contains the definition of constitutive material and boundary conditions.

### 3.1. Generation of the Geometric Model

**Figure 2:**
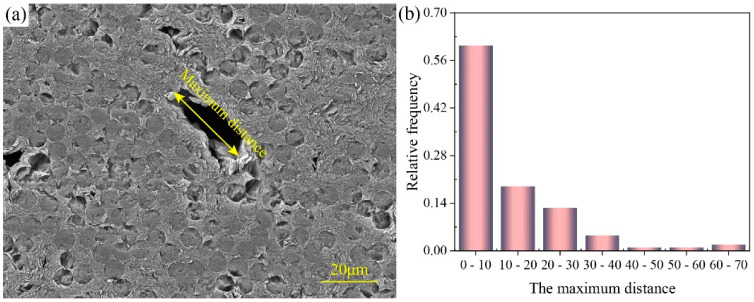
(**a**) The microstructure of UD C/C composites, and (**b**) the size distribution of the maximum distance in a pore morphology.

In UD C/C composites, the fiber size and distribution are the same in the cross-section perpendicular to the fiber axis. According to the previous study [30], the pore is elongated and parallel to the fiber axis. Thus, this paper uses the 2D RVE model based on the assumption that the pore structure is almost the same in the cross-section perpendicular to the fiber axis. Figure 2a gives the microstructure of UD C/C composites, where the maximum distance is the distance between the farthest two points. It is observed in Figure 2b that the maximum distance in pore morphology is mainly within the range of 30 μm using the image-processing technology. Meanwhile, the average maximum distance is 12.32 μm by measurement. Therefore, the pores with the maximum distance of below 30 μm are considered. 

**Figure 3:**
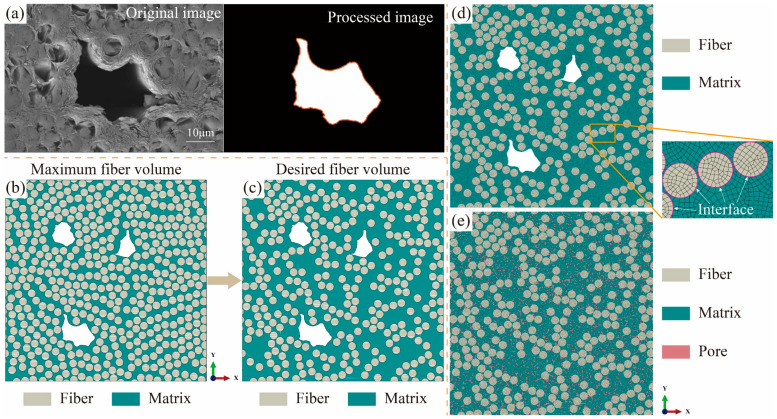
(**a**) The original SEM image and image-processed pore profile. The RVE models with (**b**) the maximum fiber volume fraction and (**c**) the desired fiber volume fraction based on the modified random fiber removal algorithm. Meshed models with (**d**) irregular pores and (**e**) elementary pores, where the subgraph presents interfacial regions between the fiber and matrix.

In Figure 3a,b, the pore profile is extracted using image-processing technology, and then the numerical realization of the pores is finished by the Python script of the 2020a Abaqus software. Before numerical modeling, the model size is required to be determined first by balancing the calculation accuracy and efficiency. According to the reported literature [18,20,21,31], UD RVE models with different fiber shapes and matrix voids have been conducted, in which the width of RVE is about eight times the fiber diameter. In addition, we use the extreme condition containing the most complex pores and the most severe pore clustering to investigate the effect of the model size based on the previous work. The results show that the model size of 175 μm × 175 μm is appropriate considering the fiber diameter of 7 μm in this paper. In the RVE domain, pores are generated using the image information, and fibers are obtained by the modified random fiber removal algorithm [32]. This algorithm is completed in two steps. In the first step, the RVE model with the maximum fiber volume fraction is established using the nearest neighbor algorithm in Figure 3b. In the second step, some fibers from the first-step RVE model are randomly removed to obtain the RVE model with the desired fiber volume fraction, as shown in Figure 3c. Figure 3d presents the meshed RVE model with the desired fiber volume fraction.

Because the factors, including pore locations, clustering, and shapes, are variable and are difficult to handle in UD C/C composites, the RVE model with elementary pores is developed to verify the whole computational framework, as shown in Figure 3e. THe random choosing of some elements from the matrix as pores is conducted by the Python script in this model. At the same time, the pore property is assigned to these elements. It is worth noting that both the matrix and fiber regions of the two RVE models are discrete first-order elements with reduced integration (CPS4R, in ABAQUS). Enhanced hourglass control technology is employed to avoid the distortion of some elements during the simulation. The elementary type of interface is COH2D4 in the subgraph of Figure 3d. The mesh size will be discussed in Section 3.

### 3.2. Constitutive Material 

To predict the nonlinear mechanical behavior of composites, the constitutive material of the fibers, matrix, and interfaces needs to be defined, respectively. According to experimental observations from Figure 1, fibers are intact under the transverse tension, compression, and shear for UD C/C composites. Therefore, fibers are considered a transverse isotropic linear elastic material without damage in this paper. 

For the damage behavior of the matrix, the strain-based Hashin law, as a widely used failure initiation, is adopted to serve as the matrix failure initiation [33]. 

For the tension ($\varepsilon_{11}^{m}+\varepsilon_{22}^{m}>0$):

(2) $$F_{mt}={\left(\frac{\varepsilon_{11}^{m}+\varepsilon_{22}^{m}}{X_{T}^{m}/E_{m}}\right)}^{2}+{\left(\frac{1}{X_{S}^{m}/G_{12}^{m}}\right)}^{2}\left({\left(\varepsilon_{12}^{m}\right)}^{2}-\frac{E_{m}^{2}\varepsilon_{11}^{m}\varepsilon_{22}^{m}}{{\left(G_{12}^{m}\right)}^{2}}\right)$$

For the compression ($\varepsilon_{11}^{m}+\varepsilon_{22}^{m}<0$):

(3) $$F_{mc}={\left(\frac{E_{m}\varepsilon_{11}^{m}+E_{m}\varepsilon_{22}^{m}}{2X_{S}^{m}}\right)}^{2}+\left(\frac{\varepsilon_{11}^{m}+\varepsilon_{22}^{m}}{X_{C}^{m}/E_{m}}\right)\left[{\left(\frac{X_{C}^{m}}{2X_{S}^{m}}\right)}^{2}-1\right]+{\left(\frac{\varepsilon_{12}^{m}}{X_{S}^{m}/G_{12}^{m}}\right)}^{2}$$

where *ε* is the normal strain. $X_{T}^{m}$, $X_{C}^{m}$, and $X_{S}^{m}$ are the tensile, compressive, and shear strengths of the matrix, respectively. *E_m_* and $G_{12}^{m}$ denote the elastic and shear moduli. In this paper, $X_{T}^{m}$, $X_{C}^{m}$, $X_{S}^{m}$, *E_m_*, $G_{12}^{m}$ are 12.1 MPa, 47.9 MPa, 19.1 MPa, 10 GPa, and 3.7 GPa, respectively [34,35]. For the fiber, the transverse elastic modulus is 15 GPa, and the Poisson’s ratio is 0.07 [32]. 

To capture stiffness recession after the failure initiation, an exponential formula is used, as shown in Equation (4) [36], where *d* lies in the range of 0 (no damage) to 1 (element broken).

(4) $$d_{i}=d_{\max}-d_{\max}\exp(-F_{m}/10e)$$

where *d_max_* of 0.95 is given, *e* is the base of the natural logarithm. To improve the convergence, a viscous regularization scheme given by Duvaut–Lions viscosity is used [16], as shown in Equation (5).

(5) $${\dot{d}}^{v}=\frac{1}{\eta }(d-d^{v})$$

where ${\dot{d}}^{v}$ is the regularized damage variable, *η* is the viscosity coefficient. By discretion, Equation (5) can be rewritten as [16]:

(6) $$d^{v}{|}_{t_{0}+\Delta t}=\frac{\Delta t}{\eta +\Delta t}d{|}_{t_{0}+\Delta t}+\frac{\eta }{\eta +\Delta t}d^{v}{|}_{t_{0}}$$

(7) $$\frac{\partial d^{v}}{\partial d}=\frac{\Delta t}{\eta +\Delta t}$$

Thus, the regularized Jacobian matrix is calculated by:

(8) $$\left[\frac{\partial \sigma }{\partial \varepsilon }\right]=\left[C^{d}\right]+\left[\frac{\partial C^{d}}{\partial d^{v}}\right]:\left\{\varepsilon \right\}\times \frac{\partial d}{\partial \varepsilon }\times \frac{\Delta t}{\eta +\Delta t}$$

where $\left[\frac{\partial C^{d}}{\partial d^{v}}\right]$ and $\frac{\partial d}{\partial \varepsilon }$ are

(9) $$\left[\frac{\partial C^{d}}{\partial d^{v}}\right]=\left[\begin{array}{lll} -C_{11} & -C_{12} & 0\\ -C_{21} & -C_{22} & 0\\ 0 & 0 & -C_{44} \end{array}\right]$$

(10) $$\frac{\partial d}{\partial \varepsilon }=\frac{\partial d}{\partial F_{mi}}\times \frac{\partial F_{mi}}{\partial \varepsilon }$$

**Figure 4:**
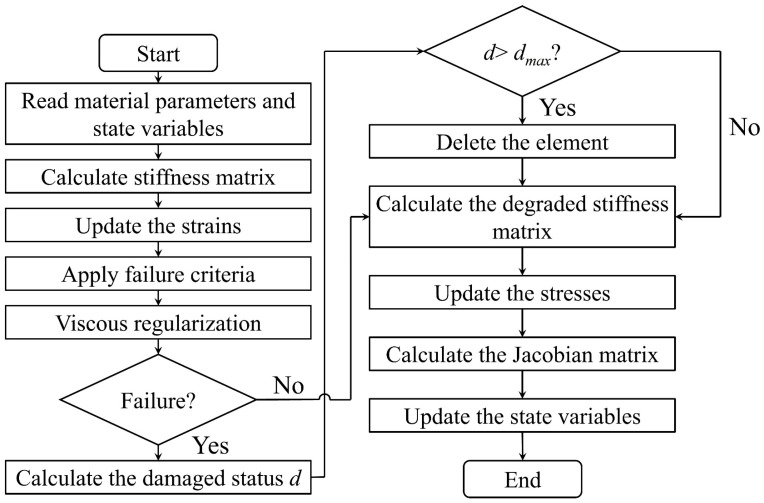
Flow chart of the damage simulation for the UD C/C composites based on the UMAT subroutine.

The above calculation of the damage initiation and evolution of the matrix is completed using the user subroutine UMAT. The specific flow is presented in Figure 4.

For the interface between the fiber and matrix, a cohesive model is utilized. The quadratic nominal stress criterion is for failure initiation. The B-K method is employed to define the interfacial softening stage [37,38]. The interfacial penalty stiffness is 10^8^ N/mm^3^, the normal strength is 12 MPa, the shear strength is 3.5 MPa, the critical toughness is 123 J/m^2^, and the coefficient for the B-K method is 2 [34,39].

### 3.3. Boundary Conditions

**Figure 5:**
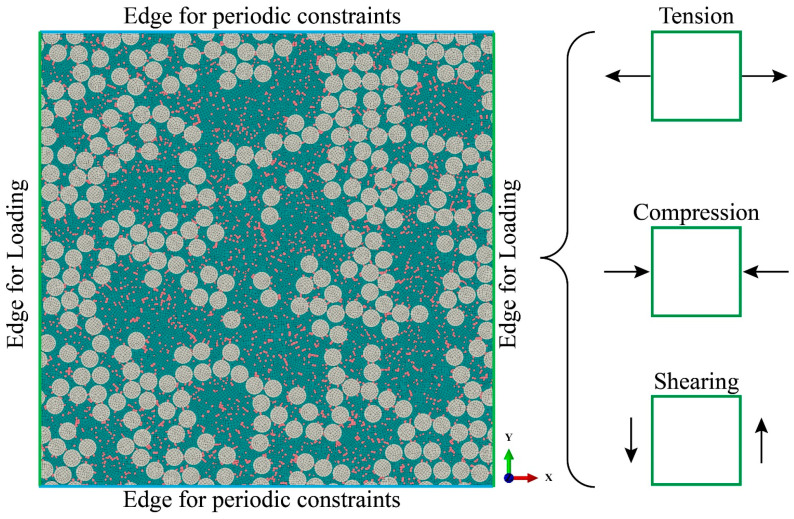
Illustrations of the boundary condition for the RVE model, which contains periodic constraints and the loading directions of tension, compression, and shearing.

This paper focuses on transverse tension, compression, and shearing. Therefore, the load is imposed on the two edges along the *x* direction while the other two edges are constrained by the periodical boundary condition, as shown in Figure 5. For the periodical boundary condition, the equations of limiting the freedom are obtained by [20]:

(11) $$\left\{\begin{array}{c} U{|}_{y=L}-U{|}_{y=0}=0\\ V{|}_{y=L}-V{|}_{y=0}=0 \end{array}\right.$$

where *L* is the RVE model size and U and V are the freedom along the *x*- and *y*-directions, respectively. In post-processing, the stress and strain are averaged for all elements in the RVE model, which is obtained by the equations $\bar{\sigma }=\int_{\Omega }\sigma dV/V$ and $\bar{\varepsilon }=\int_{\Omega }\varepsilon dV/V$. Here, *σ* and *ε* are the pointwise local stress and strain in the RVE model, and *V* is the total volume of the computational domain Ω. Results are plotted as stress–strain curves.

## 4. Mesh Sensitivity

**Figure 6:**
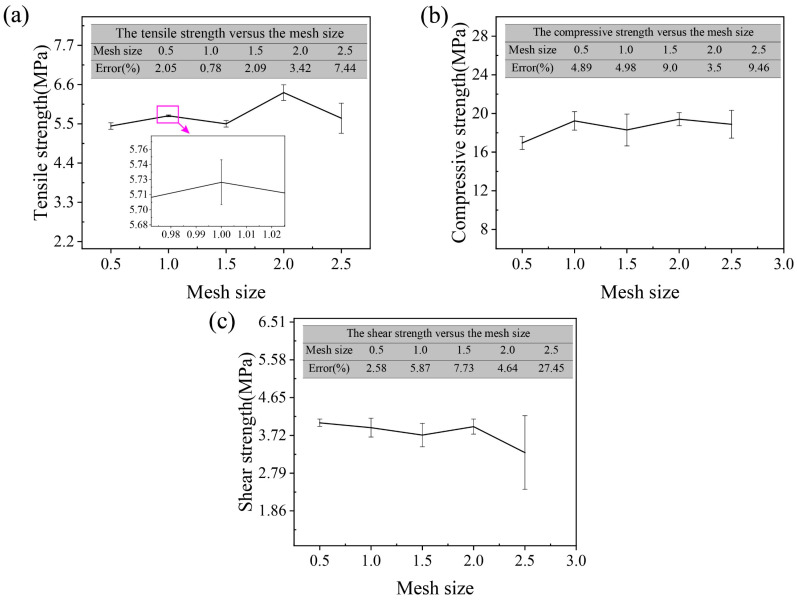
Effects of the mesh size on the (**a**) tensile strength, (**b**) compressive strength, and (**c**) shear strength of UD C/C composites.

This section studies the effect of the mesh size on the predicted results based on the RVE model with elementary pores, where the pore is considered a soft material. Taking the RVE model with a porosity of 10% as an example, Figure 6 presents the relationship between the mesh size and the strength. The relative error around the average value is calculated from the ensemble averages of the predicted strength of the relative realization number of RVE samples [21], as shown in Equation (12).

(12) $$Err=\frac{\max\left(\left|r_{i}-\frac{1}{n}\sum_{i=1}^{n}r_{i}\right|\right)}{\frac{1}{n}\sum_{i=1}^{n}r_{i}}$$

where *r* is the calculated strength and *n* is the number of independent realizations. In Figure 6, it is observed that the relative error is less than 6% when the mesh size is less than 1 μm. Thus, the mesh size of 1 μm is chosen to divide RVE models in this paper. 

## 5. Model Validation

To avoid the effects of the actual pore, the RVE model with elementary pores is used to compare against experimental results. When the porosity is 8.6%, the effective modulus *E* extracted from the global tensile stress–strain curve is 9.87 GPa based on the slope of the linear elastic regime. Here, the reported experimental result is 11.32 GPa (max: 12.45 GPa, min: 10.70 GPa) [21], which is close to the predicted result. Table 1 gives the comparison between the predicted and experimental results, where the porosity of 10% is for tension, the porosity of 8% is for compression, and the porosity of 6% is for shearing. It is found that the maximum deviation is 2.2 MPa. Therefore, the proposed computational framework is effective in predicting the elasticity and strength of UD C/C composites.

## 6. Results and Discussion

### 6.1. Effects of Porosities

**Figure 7:**
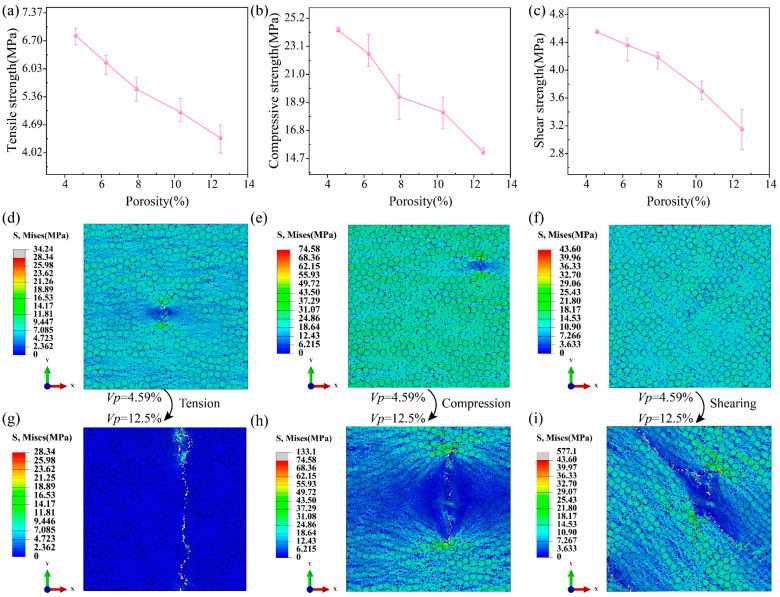
Effects of porosities on (**a**) the tensile, (**b**) compressive, and (**c**) shear strength of UD C/C composites. Stress contours of (**d**,**g**) the tension, (**e**,**h**) compression, and (**f**,**i**) shearing when the porosity is 4.59% and 12.5%, respectively.

This section investigates the effect of porosity on the strength of UD C/C composites using the RVE model with elementary pores. Figure 7a–c gives the relationship between porosity and strength. As the porosity increases, the tensile, compressive, and shear strengths will decrease. The reason is that pores will generate a stress concentration so that it is easy to reach the failure strength of the composites [40]. Moreover, if the porosity increases, the number of pores goes up, causing multi-crack propagation to be motivated more easily. When the porosity varies from 4.59% to 12.5%, the tensile, compressive, and shear strengths drop by 35.91%, 37.52%, and 30.76%, respectively. Thus, increasing the density helps to improve the mechanical properties of UD C/C composites. Figure 7d–i shows the stress contours when the complete failure happens. It is found that many scattered low-stress positions appear, which is the reason why pores have very small elastic moduli. Meanwhile, the stress concentration occurs in the front of the crack. In addition, for the porosity of 4.59%, there is no obvious crack propagation in the stress contours of Figure 7d–f. In the case of the porosity of 12.5%, the clear crack propagation path is observed, which is because the high porosity makes the matrix failure easier. The crack orientation is perpendicular to the loading direction for the tension and compression, while the angle between the crack and shear loading is about 47°. 

### 6.2. Effects of Pore Shapes

The effect of the pore shape on the strength of UD C/C composites is investigated in this section. Irregular, ellipse, and circular pores are considered, respectively. To avoid the effect of the pore location, image processing technology is applied. Firstly, irregular pores are generated in the region of the RVE model. Next, irregular pores are equivalent to ellipse and circular pores by the image processing method, respectively. Meanwhile, the locations and dimensions of the pores are obtained. Finally, RVE models with the three pore shapes are established, respectively.

**Figure 8:**
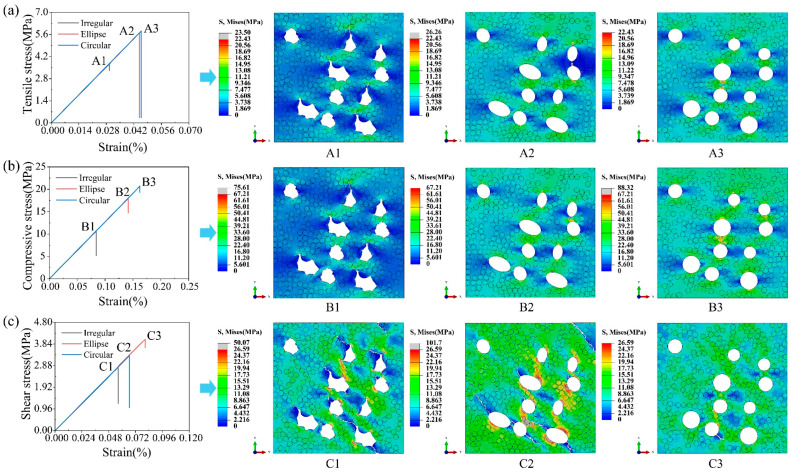
The stress–strain curve and corresponding contours of RVE models with irregular, ellipse, and circular pores under (**a**) tensile, (**b**) compressive, and (**c**) shear loading.

Figure 8 presents three stress–strain curves and stress contours. It is found that the effect of the pore shape on elastic and shear moduli of UD C/C composites is very slight. In the three cases, the RVE model with irregular pores has the smallest tensile, compressive, and shear strengths, which is because irregular pores tend to lead to stress concentrations that are up to the strength of the matrix. Thus, irregular pores easily lead to the failure of UD C/C composites. In the stress contours of Figure 8a, the peak stress in the RVE model with elliptical pores is the largest, and the stress concentration lies in the region perpendicular to the load direction. In addition, obvious crack propagation occurs in RVE models with irregular and elliptical pores. In the stress contours of Figure 8b, the length of cracks in the RVE model with irregular pores is the largest of the three cases. Besides, the ratio of the number of damaged cohesive elements to the total cohesive elements is 0.26% for the RVE model with irregular pores, 6.11% for the RVE model with ellipse pores, and 22.62% for the RVE model with circular pores, respectively. Thus, it is obtained that the crack propagation is the main failure reason of UD C/C composites with irregular pores, while the interfacial debonding is dominant in UD C/C composites with circular pores. In the stress contours of Figure 8c, it is found that the existence of pores will affect the propagation path of cracks, and the peak stress in the RVE model with elliptical pores is maximum.

### 6.3. Effects of Pore Locations

**Figure 9:**
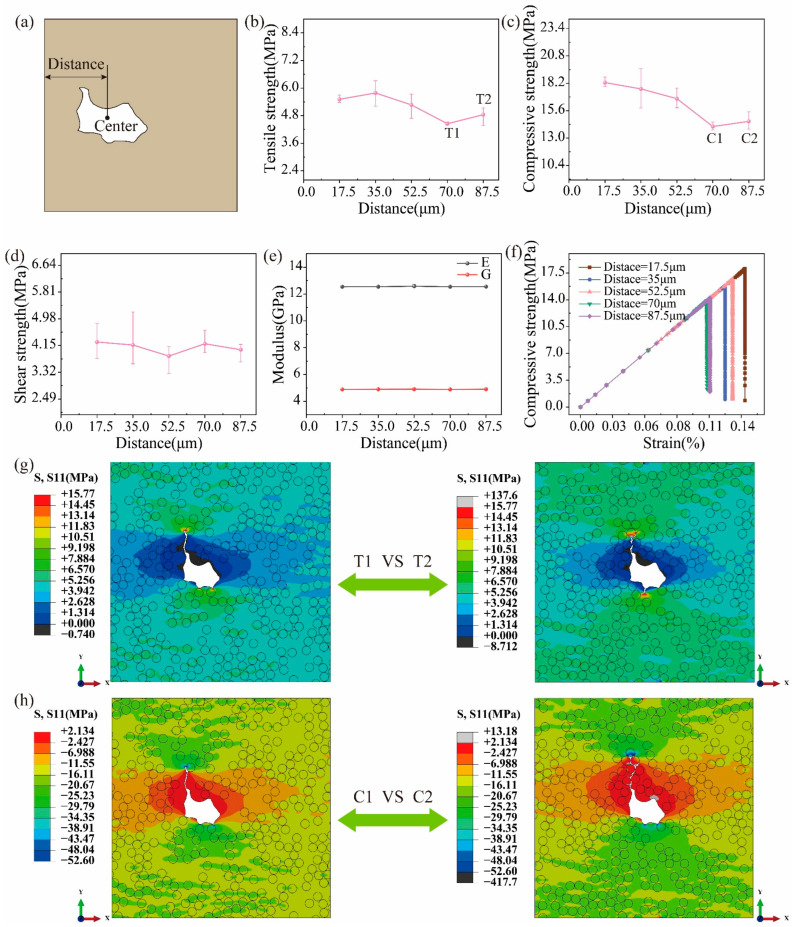
(**a**) Definition of the pore location and effects of the pore location on (**b**) the tensile, (**c**) compressive, and (**d**) shear strength. (**e**) The tensile and shear moduli versus the pore location. (**f**) The stress–strain curve for compression. (**g**) The tensile and (**h**) compressive stress contours at the max strength for the distance of 70 μm and 87.5 μm.

Plenty of irregular pores exist in UD C/C composites, as shown in Figure 1d. However, the effect of pore locations on the strengths of UD C/C composites is not studied. In this section, the control variable method is adopted, i.e., a single pore is considered. The distance between the center of the irregular pore and the left end of the RVE model is defined, as shown in Figure 9a. Figure 9b–d presents the variation curves between the distance and three strengths. It is observed that the tensile and compressive strengths decrease as the distance increases from 17.5 μm to 70 μm. When the center of the pore coincides with the center of the RVE model, the tensile and compressive strengths will increase slightly. To further analyze this phenomenon of 70 μm to 87.5 μm, the stress contour at the peak strength is obtained in Figure 9g,h. It is found that the stress concentration is larger at the position of 87.5 μm than that at the position of 70 μm for both tension and compression, which means the RVE model with the central pore has the greater resistance to the crack propagation. Thus, the pore location has a significant effect on the tensile and compressive strengths of UD C/C composites. For shearing, the strength almost remains stable when the pore location varies from 17.5 μm to 87.5 μm, as shown in Figure 9d. Besides, the effects of the pore location on the tensile and shear moduli of UD C/C composites can be neglected, which is drawn from Figure 9e. Figure 9f presents the compressive stress–strain curve of RVE models with different pore locations, where the brittle fracture is observed, and pore locations have little effect on the linear stage.

### 6.4. Effects of the Distance between Pores

**Figure 10:**
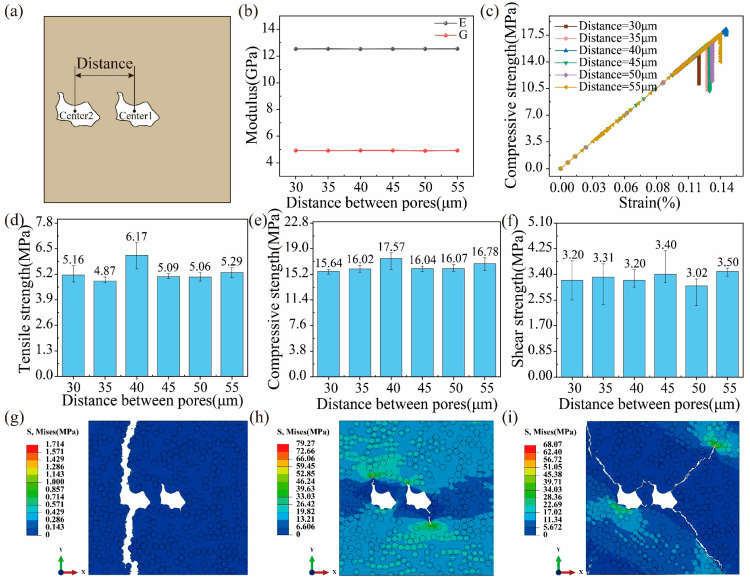
(**a**) Definition of the distance between two pores. (**b**) The tensile and shear moduli versus the pore location. (**c**) Stress–strain curves for compression. Effects of the pore location on (**d**) the tensile, (**e**) compressive, and (**f**) shear strength. Final stress contours of (**g**) tension, (**h**) compression, and (**i**) shearing when the distance is 40 μm.

In this section, the effect of interactions between two pores is investigated, where the distance between the two pores is introduced as a variable in Figure 10a. It is observed from Figure 10b that the transverse tensile and shear moduli remain unchangeable as the distance increases. Figure 10c shows the relationship between transverse compressive stress and strain for different distances. In the rising stage of the curve, the slope of all curves is almost the same, which means that the distance between pores has little effect on the transverse compressive moduli of UD C/C composites. In Figure 10d, the transverse tensile strength increases first and then decreases with an increase in the distance, where the transverse tensile strength is maximum when the distance is equal to 40 μm. Compared to the tensile strength, the compressive and shear strengths are more stable when the distance varies from 30 μm to 55 μm in Figure 10e,f. This is because the capacity of tension is very poor for the pyrolytic carbon, so the distance between pores has a more significant effect. Figure 10g–i presents the tensile, compressive, and shear stress contours with a distance of 40 μm when the computation is finished. For tension, the pore closest to the external loading end leads to crack initiation and propagation until the whole composite fails, which is because pores distributed in the load end make the criticality of the last load-bearing region exist in the end region for UD C/C composites. For compression and shearing, both pores cause crack initiation and propagation. Thus, the pore closest to the loading end dominates the tensile failure of the composite, while compressive and shear failures are related to both pores.

### 6.5. Effects of Pore Clustering

**Figure 11:**
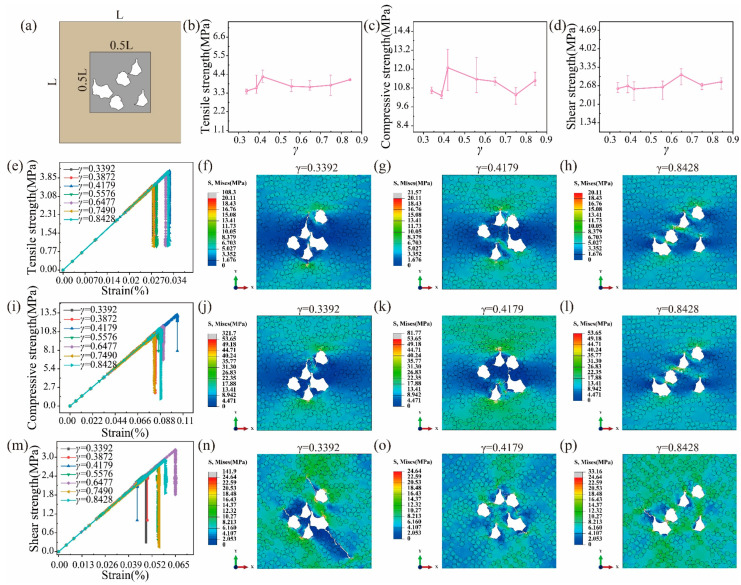
(**a**) Definition of the pore clustering and effects of the pore location on (**b**) the tensile, (**c**) compressive, and (**d**) shear strength. The stress–strain curve for (**e**) tension, (**i**) compression, and (**m**) shearing. Stress contours for (**f**–**h**) tension, (**j**–**l**) compression, and (**n**–**p**) shearing at the peak strength.

During the fabrication of C/C composites, pore clustering is observed by experimental characterization [5,41]. Thus, it is meaningful to study the effects of pore clustering on strength. To weaken the effects of pore locations, all pores are distributed in the region of 0.5*L* × 0.5*L* (*L* is the length of the RVE model), as shown in Figure 11a. Meanwhile, the average nearest neighbor index *γ* is introduced to define the degree of pore clustering, as presented in Equation (13).

(13) $$\gamma =\frac{D_{o}}{D_{e}}=\frac{\sum_{i=1}^{n}d_{i}/\left(n-1\right)}{0.5/\sqrt{(n-1)/A}}$$

where *n* is the number of pores, and *A* is the area of the RVE model. *d_i_* is the distance between a pore and its nearest neighbor pore. *D_e_* and *D_o_* are the average distance under the random and actual distribution of pores, respectively. 

In Figure 11b,c, the tensile and compressive strengths increase first, decrease, and finally increase when *γ* increases, which is because pore clustering will easily cause the local crack occur and the effects of pore locations exist. When *γ* is up to 0.8423, the effects of pore locations become dominant. With an increase in γ, the shear strength remains stable in Figure 11d. It is found from the stress–strain curve of Figure 11e,i,m that the difference in the elastic moduli for different values of *γ* can be ignored in the linear elastic stage. In Figure 11f–h,j–l,n–p, the higher the degree of pore clustering, the more obvious the crack propagation before the collapse of the UD C/C composites. 

## 7. Conclusions

In this paper, an RVE model containing the pore shape is proposed to evaluate the mechanical strength of UD C/C composites. During the calculation process, the Hashin criterion is used as the failure initiation of the matrix, and cohesive models are employed to capture the interfacial deformation between the fiber and matrix. Through studying mesh sensitivity, the mesh size is selected as one. After validating the proposed model, the effects of porosity, pore locations, the distance between two pores, pore clustering, and pore shapes are investigated. Some conclusions can be obtained as specified below.

The porosity significantly deteriorates the mechanical strength of UD C/C composites. When the porosity goes up from 4.59% to 12.5%, the tensile, compressive, and shear strengths decrease by 35.91%, 37.52%, and 30.76%, respectively. 

The effects of pore locations, the distance between two pores, and pore clustering on the shear strength, elastic, and shear moduli can be neglected. However, they have different effects on tensile and compressive strengths. For pore locations, the tensile and compressive strengths decrease as the distance increases if the pore center is not the same as the center of the RVE model. For the distance between pores, the tensile strength increases first and then decreases, while the fluctuation of compressive strength is small with an increase in the distance. For pore clustering, the tensile and compressive strengths increase first, decrease, and finally increase when the degree of the pore clustering increases.

Irregular pores make the matrix break off more easily, resulting in a significant decrease in mechanical strength, and affecting the path of crack propagation. UD C/C composites with circular pores have the highest tensile and compressive strengths.

## Figures and Tables

**Table 1:** Comparison between predicted and experimental results.

	Predicted Result	Experimental Result	Deviation
Tensile strength	5.14 MPa	3.94 ± 0.21 MPa	1.2 MPa
Compressive strength	19.94 MPa	21.0 ± 0.84 MPa	1.06 MPa
Shear strength	4.58 MPa	6.78 ± 0.7 MPa	2.2 MPa

## Data Availability

The data presented in this paper are available on request from the corresponding author.

## References

[1] Wang P., Zhang S., Li H., Kong J., Li W., Zaman W. (2014). Variation of thermal expansion of carbon/carbon composites from 850 to 2500 °C. Ceram. Int..

[2] Aly-Hassan M.S., Hatta H., Wakayama S., Watanabe M., Miyagawa K. (2003). Comparison of 2D and 3D carbon/carbon composites with respect to damage and fracture resistance. Carbon.

[3] Gillard A.P., Couégnat G., Chupin S., Vignoles G.L. (2019). Modeling of the non-linear mechanical and thermomechanical behavior of 3D carbon/carbon composites based on internal interfaces. Carbon.

[4] Rodríguez-Mirasol J., Thrower P.A., Radovic L.R. (1993). On the oxidation resistance of C/C composites obtained by liquid-phase impregnation/carbonization of different carbon cloths. Carbon.

[5] Zhang W.G., Hüttinger K.J. (2003). Densification of a 2D carbon fiber preform by isothermal, isobaric CVI: Kinetics and carbon microstructure. Carbon.

[6] Li K., Li H., Li N., Song Q., Qi L. (2022). Role of hydrogen in the dissociation of CH4 on different graphene by DFT study. Chin. J. Aeronaut..

[7] Benzinger W., Hüttinger K.J. (1999). Chemistry and kinetics of chemical vapor infiltration of pyrocarbon—VI. Mechanical and structural properties of infiltrated carbon fiber felt. Carbon.

[8] Ahmad W., Zaka U., Sonil N.I., Khan K. (2021). Introduction, production, characterization and applications of defects in graphene. J. Mater. Sci. Mater. Electron..

[9] Yoshikawa N. (2002). Modeling of Chemical Vapor Infiltration Rate Considering a Pore Size Distribution. J. Am. Ceram. Soc..

[10] Hatta H., Suzuki K., Shigei T., Somiya S., Sawada Y. (2001). Strength improvement by densification of C/C composites. Carbon.

[11] Yan K., Zhang C., Qiao S., Han D., Li M. (2011). Charpy Impact Properties of C/C and C/SiC Composties. J. Aeronaut. Mater..

[12] Li Z., Liu Z., Lei Z., Zhu P. (2021). An innovative computational framework for the analysis of complex mechanical behaviors of short fiber reinforced polymer composites. Compos. Struct..

[13] Tian Z., Yan Y., Ye J., Hong Y., Li X. (2018). An analytical constitutive model for progressive damage analysis of three-dimensional braided composites. Polym. Compos..

[14] Heidari-Rarani M., Bashandeh-Khodaei-Naeini K., Mirkhalaf S.M. (2018). Micromechanical modeling of the mechanical behavior of unidirectional composites—A comparative study. J. Reinf. Plast. Compos..

[15] Ye J., Hong Y., Liu L., Cai H., He W., Huang B., Saafi M., Wang Y., Ye J. (2022). Microscale damage evolutions in fiber-reinforced composites with different initial defects. Compos. Struct..

[16] Zhao S., Xue P. (2014). Continuum description of damage and failure of composite laminates based on viscous regularization. Multidiscip. Model. Mater. Struct..

[17] Lin S., Yang L., Xu H., Jia X., Yang X., Zu L. (2021). Progressive damage analysis for multiscale modelling of composite pressure vessels based on Puck failure criterion. Compos. Struct..

[18] Hyde A., He J., Cui X., Lua J., Liu L. (2020). Effects of microvoids on strength of unidirectional fiber-reinforced composite materials. Compos. Part B Eng..

[19] Vajari D.A., González C., Llorca J., Legarth B.N. (2014). A numerical study of the influence of microvoids in the transverse mechanical response of unidirectional composites. Compos. Sci. Technol..

[20] He C., Ge J., Cao X., Chen Y., Chen H., Fang D. (2022). The effects of fiber radius and fiber shape deviations and of matrix void content on the strengths and failure mechanisms of UD composites by computational micromechanics. Compos. Sci. Technol..

[21] Qi L., Chao X., Tian W., Ma W., Li H. (2018). Numerical study of the effects of irregular pores on transverse mechanical properties of unidirectional composites. Compos. Sci. Technol..

[22] Ali J., Berre C., Mummery P.M. (2006). Image based modelling of stress–strain behaviour in carbon/carbon composites. Energy Mater..

[23] Ai S., Fang D., He R., Pei Y. (2015). Effect of manufacturing defects on mechanical properties and failure features of 3D orthogonal woven C/C composites. Compos. Part B Eng..

[24] Xu J., Lu X., Zhu X. (2018). Effect of Random Void Defects on the Mechanical Behavior of C/C Braided Composites. Adv. Eng. Mater..

[25] Chao X., Qi L., Cheng J., Tian W., Zhang S., Li H. (2018). Numerical evaluation of the effect of pores on effective elastic properties of carbon/carbon composites. Compos. Struct..

[26] (2011). Standard Test Methods for Apparent Porosity, Liquid Absorption, Apparent Specific Gravity, and Bulk Density of Refractory Shapes By Vacuum Pressure.

[27] (2018). Standard Test Method for Monotonic Tensile Behavior of Continuous Fiber-Reinforced Advanced Ceramics with Solid Rectangular Cross-Section Test Specimens at Ambient Temperature.

[28] (2003). Advanced Technical Ceramics—Mechanical Properties of Ceramic Composites at Room Temperature—Part 2: Determination of Compression Properties.

[29] (2015). Standard Test Method for In-Plane Shear Strength of Reinforced Plastics.

[30] Drach B., Tsukrov I., Gross T., Dietrich S., Weidenmann K., Piat R., Böhlke T. (2011). Numerical modeling of carbon/carbon composites with nanotextured matrix and 3D pores of irregular shapes. Int. J. Solids Struct..

[31] Herráez M., González C., Lopes C.S., de Villoria R.G., LLorca J., Varela T., Sánchez J. (2016). Computational micromechanics evaluation of the effect of fibre shape on the transverse strength of unidirectional composites: An approach to virtual materials design. Compos. Part A Appl. Sci. Manuf..

[32] Ge J., Qi L., Chao X., Xue Y., Hou X., Li H. (2021). The effects of interphase parameters on transverse elastic properties of Carbon–Carbon composites based on FE model. Compos. Struct..

[33] Li X., Jia Y., Hong R. (2022). Comparison Between the Stress Form and Strain Form of Hashin Criteria in Progressive Failure Analysis of Composite Materials. J. Mech. Eng..

[34] Kan J. (2010). Investigation on Characterization of Micro and Meso Structures and Their Influence on Effective Properties of Carbon/carbon Composites.

[35] He X. (2009). Mechanical Properties of Kinds of Carbon Materials at Elevated Temperature.

[36] Warren K.C., Lopez-Anido R.A., Vel S.S., Bayraktar H.H. (2016). Progressive failure analysis of three-dimensional woven carbon composites in single-bolt, double-shear bearing. Compos. Part B Eng..

[37] Benzeggagh M.L., Kenane M. (1996). Measurement of mixed-mode delamination fracture toughness of unidirectional glass/epoxy composites with mixed-mode bending apparatus. Compos. Sci. Technol..

[38] Alabbad M., Vel S.S., Lopez-Anido R.A. (2022). Computational model for predicting the low-velocity impact resistance and tolerance of composite laminates. Compos. Part B Eng..

[39] Chao X., Qi L., Tian W., Yang K., Li H. (2020). Evaluation for interfacial fracture of fiber-reinforced pyrocarbon matrix composites by using a zero-thickness cohesive approach. J. Alloys Compd..

[40] Kogo Y., Hatta H., Kawada H., Machida T. (1998). Effect of Stress Concentration on Tensile Fracture Behavior of Carbon-Carbon Composites. J. Compos. Mater..

[41] Wang J., Qian J., Jin Z., Qiao G. (2006). Microstructure of C/C composites prepared by chemical vapor infiltration method with vaporized kerosene as a precursor. Mater. Sci. Eng. A.

